# Comparing the effectiveness of implementation strategies to improve liver and colon cancer screening for Veterans: protocol for a large cluster-randomized implementation study

**DOI:** 10.1186/s13012-025-01448-1

**Published:** 2025-08-09

**Authors:** Shari S. Rogal, Vera Yakovchenko, Timothy R. Morgan, Jason A. Dominitz, Heather McCurdy, Anna Nobbe, Nsikak R. Ekanem, Chaeryon Kang, Rachel I. Gonzalez, Angela Park, Jennifer Anwar, Brittney Neely, Sandra Gibson, Carolyn Lamorte, Jasmohan S. Bajaj, Heather M. Patton, Yiwen Yao, Andrew J. Gawron

**Affiliations:** 1 Center for Healthcare Evaluation, Research, and Promotion, VA Pittsburgh Healthcare System, PA Pittsburgh, USA; 2 Division of Gastroenterology, Hepatology, and Nutrition, University of Pittsburgh, PA Pittsburgh, USA; 3 Department of Surgery, University of Pittsburgh, PA Pittsburgh, USA; 4 National Gastroenterology and Hepatology Program, Veterans Health Administration, DC Washington, USA; 5 Gastroenterology Section, VA Long Beach Healthcare System, CA Long Beach, USA; 6 Division of Gastroenterology, University of California, CA Irvine, USA; 7 VA Puget Sound Health Care System, WA Seattle, USA; 8 Division of Gastroenterology, University of Washington School of Medicine, WA Seattle, USA; 9 Gastroenterology Section, VA, Ann Arbor Healthcare System, MI Ann Arbor, USA; 10 Digestive Diseases Section, Cincinnati VA Medical Center, OH Cincinnati, USA; 11 VA Northern Indiana Healthcare System, IN Fort Wayne, USA; 12 Department of Psychiatry, School of Medicine, University of Pittsburgh, PA Pittsburgh, USA; 13 Research Service, VA Long Beach Healthcare System, CA Long Beach, USA; 14 Department of Veterans Affairs, Office of Healthcare Transformation, DC Washington, USA; 15 Gastroenterology Section, Central Virginia VA Health Care System, VA Richmond, USA; 16 Division of Gastroenterology, Hepatology and Nutrition, Virginia Commonwealth University, VA Richmond, USA; 17 VA San Diego Healthcare System, CA San Diego, USA; 18 University of California San Diego, CA La Jolla, USA; 19 VA Salt Lake City Healthcare System, UT Salt Lake City, USA; 20 University of Utah, UT Salt Lake City, USA

**Keywords:** Colon cancer, Cirrhosis, Liver cancer, Screening, Fecal immunochemical testing, Surveillance

## Abstract

**Background:**

Screening for gastrointestinal (GI) cancers, specifically colorectal cancer (CRC) and hepatocellular carcinoma (HCC), is often inadequately and inequitably implemented, leading to preventable morbidity and mortality. This protocol paper describes a study designed to compare the effectiveness of external facilitation with patient navigation across hospitals in the Veterans Health Administration (VA).

**Methods:**

Two hybrid type 3, cluster-randomized trials will compare the effectiveness of patient navigation versus external facilitation for supporting HCC and CRC screening completion. Twenty-four sites will be included in the HCC trial and 32 in the CRC trial, cluster-randomizing Veterans by their site of primary care. The primary outcome of reach of cancer screening completion will be measured after intervention and during sustainment. Multi-level implementation determinants (i.e., barriers and facilitators), preconditions, and moderators will be evaluated pre- and post-intervention, using Consolidated Framework for Implementation Research (CFIR)-mapped surveys and interviews of Veteran participants and provider participants.

**Discussion:**

Comparing findings in the two trials will allow researchers to understand how implementation barriers and strategies operate differently for a one-time screening in a relatively healthy population (CRC) vs. repeated screening in a more medically complex population (HCC).

**Trial registration:**

This project was registered at ClinicalTrials.Gov (NCT06458998) on 6/13/24.

**Supplementary Information:**

The online version contains supplementary material available at 10.1186/s13012-025-01448-1.

Contributions to the literature

- This comparative effectiveness trial will compare two evidence-based implementation strategies to improve cancer screening reach.
- Comparing a patient-facing to a clinician-facing approach will offer novel insights into what works and for whom.
- This trial will assess mechanisms and heterogeneity of treatment effects to inform the science of implementation. 

## Background

Gastrointestinal (GI) cancers are among the most lethal and preventable cancers [1–4]. Specifically, colorectal cancer (CRC) and liver cancer (hepatocellular carcinoma, [HCC]), are the second and sixth leading causes of cancer death in the US [1, 3, 4]. Early cancer detection enables effective treatment, while delays lead to increased morbidity and mortality [5–8]. Because HCC most commonly occurs in people with cirrhosis, offering screening to this population is both effective and cost-effective [9, 10]. All major hepatology societies recommend HCC screening with imaging tests every six months for people with cirrhosis [11]. Similarly, because CRC death is preventable through rigorous screening programs, the US Preventive Services Task Force recommends CRC screening for all adults ages 45–75 [1]. CRC screening can start with colonoscopy or stool-based screening; abnormal stool tests must be followed with a timely colonoscopy [12, 13]. 

Despite clear evidence for HCC and CRC screening, more than 33 million people in the US who would be eligible have not had screenings [9, 11, 12, 14]. Nearly 80,000 deaths a year could be prevented if all people received the recommended GI cancer screenings [1–4]. It is therefore not surprising that CRC screening was designated as an important evidence-to-practice gap requiring implementation interventions in a recent Journal of the American Medical Association article [15]. Depending upon the provider, hospital system, and geographic area where people live, there is differential access to GI cancer screening [5–9, 16, 17]. These discrepancies suggest an urgent need to identify strategies to improve access to cancer screenings.

Implementation strategies are methods and techniques that are applied to support translation of evidence to practice in order to improve evidence-based care (e.g., cancer screening) [18, 19]. Such strategies range widely, from policy change to education, and can target patients, providers, and healthcare systems. However, no consensus exists regarding which implementation strategy is the most effective under what conditions and why [20–23]. Two of the most effective implementation strategies in health care are implementation facilitation (IF) and patient navigation. In IF, “facilitators” (implementation experts) deliver tailored provider-facing support, problem-solving tools, data, and education, in the context of a supportive relationship; IF has been rigorously tested [24–41]. Patient navigation (PN), or personalized patient support for care engagement, is supported by trials and systematic reviews, across the cancer care continuum and diverse patient populations [42–47]. However, it remains unclear how to select between these successful approaches to increase cancer screening. Therefore, this protocol outlines a study that will compare PN and IF, two evidence-based, widely used, and differently targeted strategies, addressing the question of who benefits most from patient-facing vs. provider-facing strategies to improve cancer screening. Likewise, defining the mechanisms of actions of complex strategies will aid in replication and scaling, a major implementation challenge [19].

## Methods/design

### Study aims and hypothesis

The reporting of this protocol is guided by the SPIRIT 2013 checklist [48]. The first aim of this study is to compare the effectiveness of PN and IF to increase the reach of HCC and CRC screening in the Veterans Health Administration (VA) using two hybrid type 3, cluster-randomized trials. The second aim will identify and compare how each implementation strategy addresses implementation barriers and improves patient engagement and patient-reported outcomes (PROs) using a convergent parallel mixed methods design. We hypothesize that PN will be associated with significantly increased HCC or CRC screening completion, compared to IF at 12 months post-intervention, based on literature about the effect sizes of these two approaches.

### Inclusion and exclusion criteria

VA sites are eligible if they are below the VA national median on GI cancer screening completion, where HCC screening is defined as abdominal imaging within the prior six months for Veterans with cirrhosis, and CRC screening is defined as receipt of colonoscopy within six months for Veterans with a positive stool test. We will include 24 sites in the HCC trial and 32 sites in the CRC trial. Veterans will be included and passively cluster randomized to their sites of primary care if they are 1) at least 18 years old, 2) eligible for CRC or HCC screening, and 3) enrolled in VA care in a recruited site with at least one encounter in the prior 18 months. For the HCC trial, we will define Veteran eligibility as having at least two ICD codes for cirrhosis or its complications in the electronic medical record. For the CRC trial, we will include Veterans over 45 years old who have had an abnormal fecal immunochemical test (FIT) or other screening stool test in the last 18 months. We will exclude Veterans who are not eligible for cancer screening due to receiving end of life care, defined as having a code for hospice, or who have a prior diagnosis of the cancer of interest. We will include healthcare providers or related staff working at participating VA sites who are engaged in CRC or HCC screening pathways.

### Recruitment

We will contact leadership at potential VA sites to participate in a 12-month intervention. Agreeable sites will be randomly assigned to the IF or PN arm; randomization will be stratified by site size (defined by median patients at risk of the cancer of interest) and structural characteristics (on-site vs. no on-site GI care). We will invite clinicians at participating sites to complete surveys and interviews at baseline and post-intervention to assess barriers, facilitators, preconditions, and moderators. At baseline and post intervention, a 10% subset of Veterans from the intervention sites will be mailed surveys. Post-intervention, a subset of patients will also be invited to complete follow-up interviews.

### Implementation strategies

#### Facilitation

Sites in the IF arm will participate in Getting To Implementation (GTI), a manualized intervention that guides users to select context-specific strategies with the help of a seven-step playbook, training, and external facilitation [49, 50]. GTI was adapted from Getting To Outcomes (GTO), RAND’s evidence-based program that has been successfully applied in VA and non-VA settings (e.g., Boys & Girls Clubs),[49–53] and tailored for GI cancer screening in VA using a mixed-methods, positive deviance approach (working with highly successful VA sites to identify effective strategies) [49, 50, 54, 55, 93–95]. Facilitators (one clinical expert and one evaluation expert per site) will guide site teams through the GTI steps during 1-h virtual meetings every other week for six months and provide as-needed maintenance calls for a total of 12 months of support (~ 20 h per site). Facilitators will follow best practices from VA’s Facilitation Manual to lead local providers and staff through goal setting, barrier identification, strategy selection, and iterative tests of change to improve processes of care, while engaging in interactive problem solving [24, 28, 49].

#### Patient navigation

PN sites will receive a Patient Navigation Toolkit during a one-hour introductory call with a team member who has expertise in patient navigation. The toolkit promotes three core activities with accompanying strong practices and resources: 1) using existing dashboards to identify Veterans, 2) conducting Veteran outreach to provide education, problem solve, and offer/schedule screening, and 3) documenting PN and clinical results [56]. After this introductory call, PN sites will be offered monthly opportunities to discuss progress with the experienced team member while submitting monthly tracking reports.

### Data collection

Patient data, including the primary outcome of receipt of guideline-concordant GI cancer screening, will be collected from the electronic medical records. These data will include demographic characteristics of eligible patients, rurality, area deprivation index, [57] comorbidities (organized into the Charlson score), [58] primary care provider, and relevant liver disease information (for the HCC cohort).

Site level data available from national sources include case load (number at risk and number served by VA), case mix (aggregated individual characteristics), facility complexity, region of the country, primary care panel size, and measures from the VA all-employee survey (e.g., psychological safety, servant leadership, empowerment). We will ascertain availability of resources (e.g., specialty care, on-site radiology or GI lab) from previously collected surveys. Data collection will also include pre- and post-intervention surveys and interviews with clinicians and staff from participating sites, using modified versions of the surveys shown in Table 1. We will conduct follow-up interviews with a subset of clinicians and patients.

**Table 1:** Survey assessments

**Provider data**	
Implementation determinants	Consolidated Framework for Implementation Research (CFIR) survey
Patient-centeredness	Communication & Self-efficacy in Patient Centeredness [59]
Working Alliance	Working alliance
Implementation	Acceptability, appropriateness, feasibility [60]
Burnout	Maslach burnout inventory [61, 62]
**Veteran data**	
Health-related QOL	VR-12 [63]
Care experience	Survey of Healthcare Experience of Patients
Symptom experience	Symptoms surveys [64]
Interpersonal experience	Interpersonal processes of care
Shared decision-making	Satisfaction with decision-making (CollaboRATE) [65]
Patient activation	Modified Patient Activation Measure (PAM) [66]
Health literacy	Single item literacy screener [67]

### Outcomes

The study outcomes are illustrated in Table 2 and were mapped to the RE-AIM framework [68, 69]. Each trial is powered to assess differences in reach of GI cancer screening between the two arms at 12 months from baseline. The primary outcome for HCC screening is the receipt of HCC screening by ultrasound or contrasted imaging study. We will assess patients with a diagnosis of cirrhosis at each site, and evaluate whether they had HCC screening within the prior six months. The primary outcome for CRC screening is receipt of colonoscopy after an abnormal stool test for occult blood. For the CRC trial, we will assess whether patients with an abnormal stool-based CRC screening test completed a colonoscopy within six months. We will assess Effectiveness and Maintenance as patient-level outcomes, and Adoption and Implementation (including feasibility, acceptability, appropriateness) as site-level outcomes. Fidelity to and adaptations of the implementation strategies will be tracked monthly using standard data collection forms.

**Table 2:** Outcome measures

Outcome	Specific Measure	Timepoints
**Reach** of CRC screening	CRC screening completion among patients with positive stool tests, representativeness of the population that receives screening (EMR)	Baseline & 12 months
**Reach** of HCC screening	HCC screening among Veterans with cirrhosis representativeness of the population that receives screening (EMR)	Baseline & 12 months
**Effectiveness** of screening	Tumor/polyp/lesion detection; linkage to cancer care (EMR) health benefits for rural and Black populations (EMR); change in quality of life (PRO)	Baseline & 12 months
**Adoption**	> 65% or 80% of eligible Veterans receive HCC or CRC screening, respectively (EMR)*,* reasons for non-adoption (Qu)	Baseline & 12 months
**Implementation**	How screenings and strategies are delivered	Ongoing
Feasibility	Feasibility of Intervention Measure [60] (Pr); meeting attendance, Qu	12 months
Acceptability	Acceptability of Intervention Measure [60] (Pr); Qu	12 months
Appropriateness	Intervention Appropriateness Measure (Pr); Qu	12 months
Fidelity	Whether strategies & screenings are delivered as intended (St)	Monthly
Adaptation	Changes to strategies over time (St, MADI, Qu)	12 months
Potential harm	Inequitable access (EMR), decreased engagement, trust, activation (EMR, Pt, Qu), provider mistrust, poor communication, burnout (Pr, Qu) [61, 62]	12 months
**Maintenance**	Follow-up of outcomes (EMR)	6 & 12 months post

Data Source: EMR = medical record, PRO = patient reported outcome, MADI = Model for Adaptation Design and Impact,  Pt = patient survey, Pr = provider survey, St = study team documentation, Qu = qualitative interviews

### Analysis

Results will be presented overall for each trial. We will use generalized linear mixed models (GLMMs) to assess the primary outcome of reach at 12 months. Time, intervention status, and the interaction will be modeled as fixed effects. Random effects will be included for clustering by site and Veteran. The parameters in the regression model and variance components will be estimated using the Maximum Likelihood or Restricted Maximum Likelihood. We will conduct a secondary analysis of the CRC outcome evaluating time to colonoscopy after abnormal stool test, using appropriate time-to-event models and conduct a sensitivity analysis that includes only Veterans with at least six months of follow-up after stool testing.

To understand whether and how the effectiveness of IF vs. PN varies between patients’ subgroups, we will conduct pre-specified exploratory secondary heterogeneity of treatment effect analyses. We will fit the same GLMMs as described above and include higher order interaction terms with subgroups of patients (e.g., rural vs. urban dwelling Veterans). The null hypothesis of homogenous treatment effect between Veterans within different subgroups for each outcome will be examined via interaction analysis (three-way interaction of time, treatment, and the subgroup of patients with decreased baseline rates [e.g., rural Veterans]; two-way interaction of treatment and subgroup of patients in the absence of a significant three-way interaction effect) using the same ANOVA type III Wald test based on either F- or Chi-square statistics as appropriate. In the presence of statistically significant treatment heterogeneity, we will fit a separate GLMM model for each subgroup of participants. For each outcome, the change scores from baseline to 12 months and their difference among the two treatments will be estimated using least square estimate with 95% confidence intervals for subgroups of patients.

We will assess the mechanisms of each strategy using Configurational Comparative Methods (CCM), which evaluate pathways and combinations of conditions that lead to improved PROs and cancer screening completion. Coincidence Analysis (CNA) is the CCM that we will use to incorporate both qualitative and quantitative data. Following data calibration and transformation, we will use the multi-value CNA function within the R “cna” package to identify combinations of conditions (solutions) that distinguish implementation and patient outcomes, based on the strongest parameters of fit (i.e., coverage and consistency). We will also compute a criterion of fit-robustness to avoid overfitting and to reduce model ambiguities. These findings will identify pathways through which PN and IF can address barriers to improve results.

We will evaluate the data from interviews and surveys independently, then conduct concurrent triangulation to evaluate areas where quantitative and qualitative data can be integrated, and examine convergence, expansion, discrepancy, and complementarity. We will use Rapid and rigorous qualitative data analysis coding, guided by the updated Consolidated Framework for Implementation Research (CFIR) [70]. Data will be double coded by two trained and experienced members of our team. Inter-coder reliability will be assessed using Cohen’s kappa statistic, with coding discrepancies resolved by joint critical review and revisiting until negotiated consensus is reached [71].

### Sample size and power

Power calculations were informed by pilot data, literature, and similar studies. Estimates included VA data to calculate the mean number of eligible patients per site (811 ± 387 for HCC, and 429 ± 265 for CRC), coefficient of variance of cluster sizes (0.48 for HCC and 0.631 for CRC) within-person correlation of 0.2 (for HCC), and baseline screening rates. We applied a moderate ICC of 0.03 but varied the ICC up to 0.1 in our models. Based on literature demonstrating ~ 17% improvement in cancer screening with PN and ~ 9% improvement in screening with IF, we powered the study to detect an expected difference in screening rates between the PN vs. IF arms of 0.085 [24–47].

The cps.did.binary function in the R package clusterPower [72] was modified to include coefficients of variation of the cluster sizes using simulations (average of 1000 simulated data = 0.48 for HCC; 0.631 for CRC) and account for within-person and within-site correlation and validated these calculations using simulations that allow the hierarchical clustering [73]. Varying the parameters widely (e.g., difference in effect size as low as 0.03 between arms) and conducting over 1000 simulations, the HCC models demonstrated > 85% power with 12 sites per arm, and the CRC models retained > 80% power with 16 sites per arm.

## Discussion

This research protocol addresses a critical gap in implementation science. Though implementation literature recommends that strategies be selected to address specific implementation barriers (i.e., strategies are not “one size fits all”),[21, 22, 74–77] it remains unclear how to operationalize this recommendation. This study will test whether and when PN (a one-size-fits-all, patient-facing approach) works better than working with sites to select strategies based on barriers. Comparing how these strategies work across two types of cancer screening will offer further insights into when to apply one-size-fits-all PN vs. IF, while also allowing us to identify and understand differences between a one-time, high-intensity screening in a generally healthy population (CRC) and repeated cancer screening in a more medically complex population (HCC).

There is equipoise about which strategy will work better. IF works by addressing provider and site barriers. With facilitator support, sites build an internal team, identify strengths and barriers, and select the approach that will work to improve implementation. In contrast, PN focuses on overcoming patient barriers to seeking care. Navigators work directly with patients, which can circumvent barriers of awareness, education, and buy-in. A systematic review of 25 studies found that IF improved cancer screening by an average of 5%–8%;[78] CRC screenings have been increased up to 11% using IF [32, 40]. While both IF and PN are evidence-based, they also have potential disadvantages in certain settings [27, 79–81]. PN has improved HCC and CRC screening, including colonoscopy after positive stool test [82–87]. Our study will evaluate for what type of site (specialty care availability and other organizational characteristics), what type of patient (who benefits most and least) and when (for a one-time screening vs. ongoing screening) PN or IF work better to promote screening and improve patient reported outcomes.

Precision implementation science has evolved as a sub-discipline aiming to develop empirical approaches to selecting effective implementation strategies [88]. Precision implementation promises to help healthcare systems avoid waste and reduce variation in cancer screening and follow-up [89]. However, the absence of pragmatic trials comparing the effectiveness of implementation strategies is a critical gap in implementation science that must be addressed to realize the promise of precision implementation [90–92].

### Methods to address limitations

We will assess the robustness of the conclusions using sensitivity analyses and report on all patients, any reasons for dropout, and differences in dropout by patient and site characteristics. Since our primary outcomes will be assessed using VA Corporate Data Warehouse data, we anticipate negligible missingness in the primary outcome of cancer screening completion. Non-screening will be assumed for those without confirmed screening. During the data collection, we will reduce missingness (to the extent possible) in covariates, using additional data sources with more complete data. If the missing proportion is high (e.g., > 5%), we will conduct a sensitivity analysis to assess the impact of the missing data on the primary analysis results and model the missing data mechanisms using established methods. Demographic characteristics and key clinical outcomes will be compared between the subgroup with complete vs. incomplete data, and all sensitivity analysis results will be reported. To address challenges with survey response rates, we will follow guidance about provider and patient surveys from the literature and our Veteran Advisory Board, centering research reciprocity, ensuring clarity, and minimizing survey burden. Despite our focus on US Veterans, these trials were designed to offer generalizable findings for community settings and non-Veterans. The mechanisms and comparative effectiveness of different implementation strategies are of interest beyond VA, and VA-generated implementation science methods and findings have often successfully translated to non-VA settings.

## Conclusion

In summary, these trials aim to improve the efficiency of the largest integrated US healthcare system. Because our research questions, comparators, and methods were developed in partnership with leadership, Veterans, and clinicians, the findings are expected to be actionable and implementable at-scale. These findings will offer data about how to select implementation strategies to improve cancer screening across hospitals, patient populations, and geographic settings, further informing a move towards precision implementation. 

## Supplementary Information

Supplementary Material 1.

## Data Availability

N/A.

## Abbreviations

CCM: Configurational Comparative Methods
CNA: Coincidence analysis
CRC: Colorectal cancer
FIT: Fecal immunochemical test
GI: Gastrointestinal
GLMM: Generalized linear mixed model
GTI: Getting To Implementation
GTO: Getting To Outcomes
HCC: Hepatocellular carcinoma
IF: Implementation facilitation
PN: Patient navigation
PRO: Patient-reported outcome
US: United States
VA: Veterans Health Administration
